# A diagnostic dilemma in a child with Wilson's disease

**DOI:** 10.1007/s00467-026-07186-w

**Published:** 2026-02-26

**Authors:** Dina E. Sallam, Gloria Thomas Guergues, Noha Mohsen Samak, Mohammed Eladawy, Sally Gouda Mohammed, Armia Mounir Agour

**Affiliations:** 1 Department of Pediatrics and Pediatric Nephrology, Faculty of Medicine, Ain Shams University, Cairo, Egypt; 2 Faculty of Medicine, Ain Shams University, Cairo, Egypt; 3 Department of Ophthalmology, Faculty of Medicine, Ain Shams University, Cairo, Egypt; 4 Department Pediatric Hepatology, Faculty of Medicine, Ain Shams University, Cairo, Egypt; 5 Department of Pediatrics and Pediatric Allergy, Immunology and Rheumatology, Faculty of Medicine, Ain Shams University, Cairo, Egypt

**Keywords:** AKI, Cu, Infection, Fanconi, Chelation, WD

## Abstract

**Background:**

Wilson's disease (WD) is a rare copper (Cu) metabolism disorder with toxic organ accumulation. Diagnosis challenges persist in children with atypical multisystem presentations, including uncommon kidney involvement, that may mimic autoimmune and systemic diseases.

**Clinical Presentation:**

A 10-year-old girl presented with recurrent jaundice, abdominal pain, weight loss, poor school performance, and polyuria. She had cachexia, hepatosplenomegaly, kinetic hand tremors, non-immune hemolytic anemia, and mild glomerular and tubular dysfunction. Urosepsis triggered decompensation with worsening hepatic function, acute kidney injury (AKI) with Fanconi-like tubulopathy, coagulopathy, low complements, + ve autoantibodies, and elevated IgG. Despite autoimmune and malignancy mimics, Cu studies confirmed WD; D-penicillamine and zinc achieved rapid hepatorenal and hemolysis recovery. Fibrosis regressed at 1 year despite late ocular affection.

**Conclusion:**

This case underscores diagnostic challenges of pediatric WD with multisystem involvement mimicking autoimmune and malignant disorders, emphasizing early Cu studies in multisystem jaundice. Timely chelation therapy achieved rapid improvement.

## Introduction

Wilson's disease (WD) is a rare disorder of Cu metabolism caused by *ATP7B* gene mutation, resulting in impaired Cu excretion and toxic accumulation in multiple organs [1]. It is a multisystem disease with diverse manifestations, mainly hepatic or neuropsychiatric. It can mimic autoimmune conditions, particularly autoimmune hepatitis (AIH), with nonspecific features, creating a diagnostic challenge [2, 3]. Kidney affection in WD is less common with variable morbidity [4].

## Case presentation

We report a 10-year-old girl, born to consanguineous parents presenting with a 3-month history of recurrent anicteric jaundice, abdominal pain, weight loss, poor academic performance, and polyuria with no changes in urine or stool color. Examination showed cachexia, normal blood pressure (50th percentile), mild hepatosplenomegaly, audible cardiac hemic murmur, kinetic hand tremors, with normal ocular examination.

 Laboratory investigations (Table 1) initially showed impaired kidney function, hypokalemia, elevated transtubular potassium gradient (TTKG), albuminuria, and normal anion gap metabolic acidosis, mild aminotransaminase elevation, hyperbilirubinemia, thrombocytopenia, non-immune hemolysis, high IgG, + ve ASMA, low C3 and C4. Imaging showed mild hepatosplenomegaly and lymph node enlargement near the liver's portal vein (porta hepatis lymphadenopathy) with normal kidneys. At this stage, the patient was clinically stable.

**Table 1:** Laboratory data at initial presentation, diagnosis and follow up

Variable data	Initial presentation (Weight = 26 kg, Height = 128 cm)	At time of decompaction (Weight = 25 kg, Height = 128 cm)	Follow-Up after 3 months of therapy (Weight = 30 kg, Height = 129 cm)	Reference range
**Liver function tests**				
ALT (U/L)	48	9	28	7–45
AST (U/L)	112	100	33	8–40
Total bilirubin (mg/dL)	6.4	3	0.5	0.2–1.2
Direct bilirubin (mg/dL)	3.4	1.1	0.1	0–0.3
Albumin (g/dL)	3.0	2.5	5.7	3.5–5.2
Total protein (g/dL)	6.1	4.4	7.9	6–8
PT (sec)	29	20.6	13	11–15
PTT (sec)	54	33	24	25–35
INR	2.2	1.59	1.0	0.8–1.2
Alkaline phosphatase (U/L)	79	131	70	40–150
Gamma GT (U/L)	96	103	20	10–50
**Serum electrolytes, kidney function tests**				
Serum sodium (mmol/L)	135	136	135	135–145
Serum potassium (mmol/L)	3.1	2.6	3.8	3.5–5.0
Serum magnesium (mg/dL)	1.9	2.0	2.2	1.7–2.3
Serum phosphorus (mg/dL)	2.6	2.0	5.3	2.5–4.5
Serum uric acid (mg/dL)	1.5	1.0	4	2–6
Serum creatinine (mg/dL)	1.71	2.67	0.54	0.5–1.0
eGFR by Schwarz formula	31	20	99	> 90 (mL/min/1.73 m^2^)
Blood urea (mg/dL)	35	55	8	10–40
Serum pH	7.30	7.23	7.38	7.35–7.45
Serum HCO3 (mEq/L)	18	12	24	21–29
24 h urinary protein (mg/day)	200	318	35	< 150
Albumin creatinine ratio (mg/g)	150	280	20	30–300
24 urinary volumes (mL/day)	2500	2800	1200	800–2000
Urine output (mL/kg/hour)	4	4.6	1.6	0.5–2 mL/kg/hr
Urine specific gravity	1015	1010	1025	1.005–1.030
Urine PH	5.9	6.5	5.0	4.5–8.0
Urine glucose	Trace	+ 1	Nil	Nil
TTKG	10	–-	6.5	6–10
**Viral markers**				
CMV IgG (AU/mL)	Negative	Negative	Negative	Negative
EBV IgG	Negative	Negative	Negative	Negative
Hematological markers				
Total leukocyte count (× 10^3^/µL)	4.1	2.8	9.1	4–11 × 10^3^ (/µL)
Hemoglobin (g/dL)	8.1	5.3	10.5	12.1–15.1 g/dL
Mean corpuscular volume (fL)	95	120	89	80–100 fL
Reticulocyte count %	2.61	10	2.1	0.5–2.5%
Platelet (× 10^3^/µL)	60	40	162	150,000–450,000
TIBC (µg/dL)	142	120	333	250–450
Transferrin saturation (%)	92	95	20	15–50
Immunological markers				
ANA	Negative	Negative	Negative	Negative
Anti DNA Ab	Negative	Negative	Negative	Negative
C3 (mg/dL)	78	44.2	120	90–180
C4 (mg/dL)	< 8	< 8	30	10–40
ASMA	Positive	Positive	Negative	Negative
Serum IgG (mg/dL)	3500	4100	750	700–1600
Serum IgM (mg/dL)	131	150	70	40–230
Coombs test	Negative	Negative	Negative	Negative
**Others**				
Random blood sugar (mg/dL)	100	80	110	Up to 180
CRP (mg/L)	< 5	320	< 5	< 5
LDH (U/L)	300	681	130	140–280
Cu Metabolism	At the time of diagnosis and confirmation* across laboratories	Biochemical response 3 weeks post chelation	Biochemical response one year post chelation	Reference range
Serum Ceruloplasmin (mg/dL)	8.6–8.8*	29.9	31	20–40
24 h Urinary Cu (µg)	1330–1337*	153	98	< 100

Two days later she developed fever, worsening pallor, jaundice, tachycardia, hypotension, polyuria, ascites, and loin tenderness. Repeat investigations showed declining eGFR, hypokalemia, hypophosphatemia, hypouricemia, normal anion gap metabolic acidosis, low urine specific gravity, albuminuria, and glucosuria with normoglycemia, hyperbilirubinemia, transaminase elevation, pancytopenia, non-immune hemolysis, and coagulopathy. Abdominal imaging revealed hepatosplenomegaly, ascites, thickened peritoneal reflections, mesenteric vascular congestion, persistent porta hepatis lymphadenopathy, bilateral hyperechogenic kidneys and grade 1 nephropathy, no nephrocalcinosis or nephrolithiasis. Fibroscan confirmed advanced hepatic fibrosis.

Acute liver failure (ALF) was considered, with differentials including malignancy, AIH, systemic lupus erythematosus (SLE), immune overlap hepatitis, and WD. She received vasopressors, blood transfusions, fluids, and empirical broad-spectrum antibiotics, followed by dramatic improvement within 4 days, though hemolysis persisted. In the context of ongoing hemolysis and multisystem involvement, she was empirically given a 3-day course of pulse steroid for suspected catastrophic SLE with overlapping lupus hepatitis, later stopped after urine culture confirmed Gram -ve urosepsis responsive to cephalosporins, implicating urosepsis as the cause.

Improvement in clinical and laboratory findings made malignancy, AIH, and SLE less likely despite + ve ASMA. Results of 24-h urinary Cu excretion and serum ceruloplasmin measurement confirmed WD, repeated in different laboratories for confirmation [5]. Screening of siblings biochemically was negative. Early chelation with D-penicillamine and zinc sulfate, oral potassium, and culture-guided antibiotics led to rapid recovery within 3 weeks, though ASMA + ve, reflecting reactive autoimmunity.

At 3-month follow-up, patient showed sustained improvement, with normalization of all her laboratory parameters and -ve ASMA. At that time, liver biopsy revealed chronic hepatitis with significant inflammation and fibrosis, but no Cu deposition, prompting D-penicillamine dose reduction.

At 1 year post therapy, new dysarthria and Kayser–Fleischer (K-F) rings appeared on examination, yet fibrosis regressed on Fibroscan, with no additional organ involvement, requiring no therapy changes.

## Discussion

This case illustrates important facets of pediatric WD, including diagnostic challenges, multisystem involvement with rare but significant kidney affection.

### Diagnostic dilemma and overlapping syndromes

This was a complex presentation, with a self-limited hepatitis-like illness, followed by ALF, precipitated by urosepsis, unmasking latent Cu toxicity and multiorgan dysfunction. Diagnosis was challenging due to overlap with AIH, SLE, and malignancy. Malignancy was considered due to organomegaly, porta hepatis lymphadenopathy, pancytopenia, persistent fever, and high IgG. AIH was suspected based on + ve ASMA, elevated transaminases, IgG; SLE owing to pancytopenia, low C3 and C4, high IgG, and multisystem involvement.

Malignancy was excluded by resolution of cytopenia after infection control. SLE was excluded using 2019 EULAR/ACR criteria [6], given -ve anti-dsDNA and ANA antibodies, absence of mucocutaneous features, transient low C3 and C4, and lack of persistent multisystem affection. AIH was unlikely due to normalized IgG, -ve viral serology, and clinical improvement, despite + ve ASMA.

Evaluation showed these findings were secondary. Transient hypocomplementemia reflected impaired hepatic synthesis during ALF, as complement is hepatic protein derived [7]. Fever, elevated IgG, porta hepatis lymphadenopathy, and pancytopenia were due to urosepsis rather than autoimmunity. Autoantibodies may occur in WD, chronic liver disease, or healthy children, explaining the + ve ASMA [2].

### Clinical and biochemical hallmarks of WD in children

WD manifests differently by age; young children present often with hepatic and hematological features, whereas older children and adults show neuropsychiatric symptoms [1, 8]. Our patient exhibited severe hepatic WD profile with modest aminotransferase elevation, marked hyperbilirubinemia, and low alkaline phosphatase [1, 8].

Diagnosis was established by Leipzig score > 4, based on neuropsychiatric signs, non-immune hemolytic anemia, elevated 24-h urinary Cu, and low serum ceruloplasmin, necessitating prompt therapy initiation [5, 8].

Kidney involvement: is less common than hepatic or neurologic manifestations, yet Cu exerts direct toxic damage on renal tubules, renal tubular acidosis, ranging from mild electrolyte and acid–base disturbances to Fanconi syndrome. These defects predispose to nephrolithiasis, nephrocalcinosis, bone disease, and AKI, which increases mortality. AKI arises from hypoperfusion, hemolysis-related tubular casts, or D-penicillamine-induced glomerulopathy [4]. Our patient had combined glomerular and proximal tubular dysfunction with AKI, hypokalemia with high TTKG, glucosuria with normoglycemia, hypouricemia, non–anion gap acidosis, albuminuria, and polyuria. During urosepsis, findings progressed to Fanconi-like syndrome, worsening eGFR and hypokalemia, hypophosphatemia, profound hypouricemia, non-anion gap metabolic acidosis, high urine pH, polyuria, albuminuria and glucosuria despite normoglycemia. After 3 months, laboratory data normalized, demonstrating reversibility with timely treatment intervention. Due to limited resources, specific markers such as tubular phosphate reabsorption, fractional excretion of uric acid and urinary beta 2 microglobulin were not available.

Ocular findings: Cu deposits in Descemet’s membrane causing K-F rings are a hallmark of WD, occurring in 95% with neurological affections and 50% without. In children with hepatic disease, K-F rings are absent or difficult to detect [9]. This explains our observation initially, despite subtle neuropsychiatric features, while hepatic involvement predominated. Notably, K-F rings appeared 1 year post therapy, despite improved hepatic Cu and fibrosis. Therefore, absence of K–F rings does not exclude pediatric WD [5]. Ocular manifestations may emerge later in the disease course, and warrant ophthalmologic monitoring.

### Therapeutic response and reversibility

Treatment of WD in children targets Cu reduction and organ protection, requiring early pharmacologic therapy with chelators such as D-penicillamine and zinc to inhibit intestinal absorption [8]. In our patient, urosepsis was managed with cefotaxime (75 mg/kg/8 h) for 10 days, guided by culture sensitivity. Therapy included Cu restriction, D-penicillamine (20 mg/kg/day, reduced to 15 mg/kg/day after 3 months), zinc sulfate (1 mg/kg/day) [8], oral potassium (1 mmol/kg/day) for hypokalemia. This led to rapid clinical improvement. Monitoring comprised; quarterly: 24-h urinary Cu, serum ceruloplasmin, liver and kidney function, acid–base and electrolyte status, and hematological parameters; every 6 months: slit-lamp examination and Fibroscan; annually: liver biopsy to assess fibrosis.

New dysarthria and K-F rings appeared at 1 year. We recommend ongoing six-monthly biochemical and hematological assessment, slit-lamp examination, and Fibroscan, with annual liver biopsy. Genetic testing and counseling for relatives is needed.

Family screening biochemically by serum ceruloplasmin and urinary Cu excluded WD in siblings. Though sequencing of the *ATP7B* gene was recommended for the patient and her siblings; we could not perform it due to financial issues.

## Conclusion

This case highlights diagnostic challenges of pediatric WD, where urosepsis precipitated decompensation, that mimicked malignancy and autoimmune disease. Though kidney involvement in WD is uncommon, it is often heterogeneous and subtle, underscoring the need for monitoring of kidney function before and during treatment. Comprehensive assessment remains essential for diagnosis and intervention for therapeutic response and reversibility.

## Summary

### What is new?

- A previously healthy girl with unrecognized WD had urosepsis, that triggered fulminant WD, mimicking autoimmune disease and malignancy. Biochemical study confirmed WD. Early Cu chelation reversed organ damage.

## Data Availability

Available on request**.**
